# Direct dacryoendoscopic probing and endonasal dacryocystorhinostomy for treatment of acquired dacryocystocele: A case series

**DOI:** 10.1097/MD.0000000000043159

**Published:** 2025-07-18

**Authors:** Muhammad Abumanhal, Kinga Yo, Miho Enoki, Yuki Wasai, Yasuhiro Takahashi

**Affiliations:** a Department of Oculoplastic, Orbital & Lacrimal Surgery, Aichi Medical University Hospital, Nagakute, Aichi, Japan; b Department of Otorhinolaryngology-Head and Neck Surgery, Aichi Medical University School of Medicine, Nagakute, Aichi, Japan; c Department of Ophthalmology, Ogori Daiichi General Hospital, Yamaguchi, Japan.

**Keywords:** acquired dacryocystocele, dacryoendoscope, direct probing, endonasal dacryocystorhinostomy

## Abstract

**Rationale:**

Acquired dacryocystocele is a rare condition in adults and is often associated with distal and proximal lacrimal drainage obstructions. While proximal obstruction has generally been presumed to be functional, this assumption has not been definitively confirmed in previous studies. In this case series, we report for the first time the use of direct dacryoendoscopic probing as both a diagnostic and therapeutic adjunct to endonasal dacryocystorhinostomy (DCR) in the management of acquired dacryocystocele.

**Patient concerns:**

Three patients (1 male and 2 females; age, 66–88 years) presented with unilateral epiphora and medial canthal swelling. Symptom duration ranged from 2 weeks to 2 years. One patient exhibited bluish discoloration over the medial canthus; another had a history of nasal surgery and concomitant frontal sinus mucocele with sinusitis.

**Diagnoses:**

All patients were diagnosed with acquired dacryocystocele based on clinical examination and imaging. Dacryoendoscopy confirmed complete mechanical common canalicular obstruction in each case.

**Interventions:**

Each patient underwent endonasal DCR combined with intraoperative direct dacryoendoscopic probing to relieve the canalicular obstruction, followed by bicanalicular intubation.

**Outcomes:**

All patients achieved complete symptom resolution and demonstrated a widely patent rhinostomy on endoscopic follow-up at 6 months. No intraoperative or postoperative complications or recurrences were observed.

**Lessons:**

This series suggests that mechanical common canalicular obstruction may be a key mechanism in the pathogenesis of acquired dacryocystocele. Incorporating direct dacryoendoscopic probing during DCR enables real-time identification and treatment of proximal obstruction, potentially improving surgical success and minimizing recurrence.

## 1. Introduction

Dacryocystocele is a notable cystic enlargement of the lacrimal sac, typically filled with secretions from its epithelial lining, which contains goblet cells.^[1,2]^ While congenital dacryocystocele is more common, the adult-acquired form is rare.^[2]^ Acquired dacryocystocele results from both distal nasolacrimal duct obstruction and proximal common canalicular obstruction.^[2,3]^ Although the most proposed mechanism of the common canalicular obstruction is functional obstruction at the valve of Rosenmüller,^[2,4]^ none of the previous studies have proven this mechanism.

The treatment of acquired dacryocystocele includes external or endonasal dacryocystorhinostomy (DCR), with or without silicone tube placement or dacryocystectomy.^[4–6]^ None of the previous studies had used a dacryoendoscope to confirm mechanical or functional common canalicular obstruction and avoid blind probing and silicone tube placement for treatment of common canalicular obstruction. In this case series, we present 3 cases of adult dacryocystocele treated with direct dacryoendoscopic probing and endonasal DCR.

## 2. Methods

This study was conducted in accordance with the tenets of the Declaration of Helsinki and its later amendments. This study protocol was reviewed, and the need for approval was waived by the institutional review board of our university hospital, based on the ethical guidelines for medical and health research involving human subjects established by the Japanese Ministry of Education, Culture, Sports, Science, and Technology and the Ministry of Health, Labor, and Welfare, because this was a retrospective study including a small number of patients. Written informed consent was obtained from all patients for publication of clinical course and from Case #2 for publication of an identifiable face photo.

We retrospectively reviewed the medical records of all patients with acquired dacryocystocele, who were treated with direct dacryoendoscopic probing and endonasal DCR from January 2024 to September 2024. Data on age, sex, affected side, past medical history, duration of symptoms, presence or absence of bluish swelling in the medial canthal region, pathological results of a lacrimal sac specimen, and culture test for lacrimal sac content were collected. Axial and coronal orbital computed tomographic (CT) images with bone and soft tissue window algorithms were obtained from all patients. Presence or absence of concomitant nasal pathology was confirmed on CT images.

**Figure 1.**
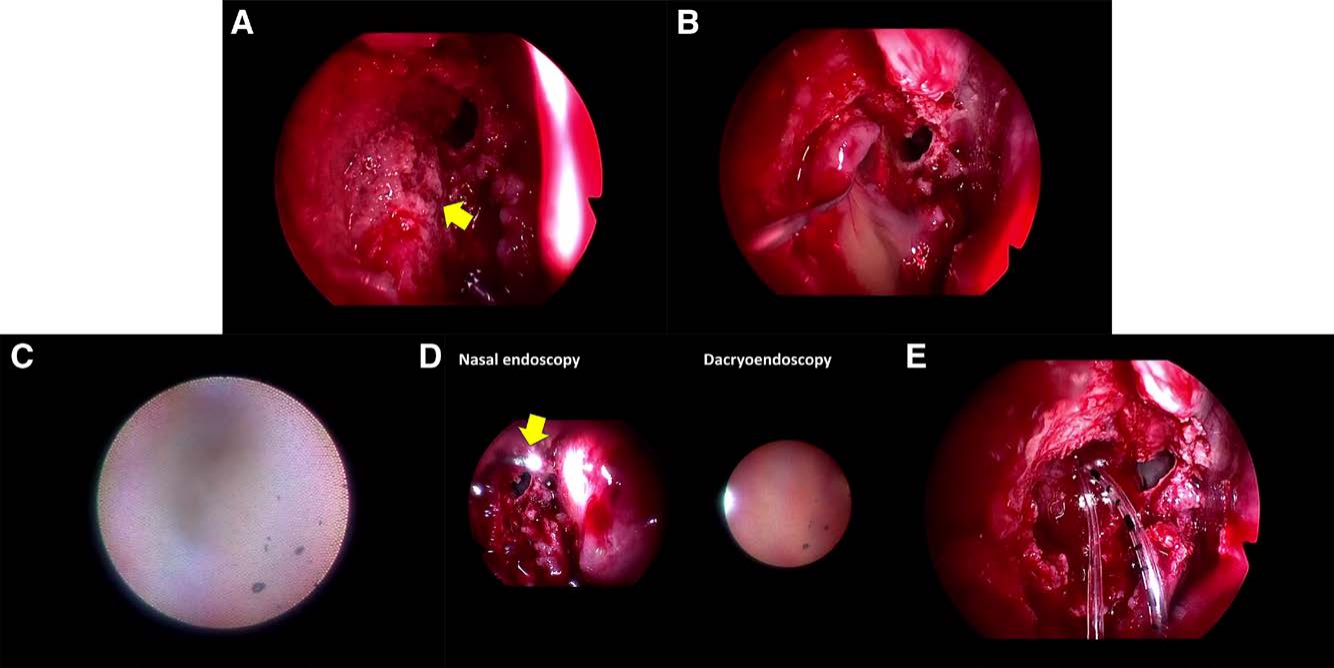
Intraoperative photos in Case #2. (A) After opening a bony window, the lacrimal sac was exposed (arrow). (B) The lacrimal sac was opened, and lacrimal sac content was drained. (C) Common canalicular obstruction was found under dacryoendoscopic examination. (D) After direct dacryoendoscopic probing, the tip of the dacryoendoscope (arrow) was confirmed using a nasal endoscopy. (E) A bicaanlicular lacrimal tube was inserted.

Surgery was performed under general anesthesia by the author (YT) in all cases. In the case involving a concomitant frontal sinus mucocele, the procedure was performed in collaboration with an otorhinolaryngologist (KY). A 4 mm rigid nasal endoscope with a 30-degree viewing angle was used during the endonasal DCR. After making a posteriorly-based nasal mucosal flap anterior to the axilla of the middle turbinate, the flap was excised. The bone overlying the lacrimal sac was removed using either an ultrasonic aspirator (Sonopet UST-2001, Stryker Japan K.K., Tokyo, Japan) or a high-speed drill (Fig. 1A). The lacrimal sac was then opened, drained, and its contents were sent for culture (Fig. 1B). The medial wall of the lacrimal sac was removed to achieve wide marsupialization. Following a 2-snip punctoplasty, a dacryoendoscope (Ruido Fiberscope; FiberTech Co., Tokyo, Japan) was inserted into the lacrimal punctum to examine the internal anatomy of the lacrimal drainage system. Saline was flushed through the system, allowing visualization of the upper and lower canaliculi, common canaliculus and lacrimal sac, confirming the presence of a common canalicular obstruction (Fig. 1C). After identifying the obstruction site and visualizing the nasal cavity with the dacryoendoscope, the appropriate location was confirmed using nasal endoscopy. Direct dacryoendoscopic probing was then performed to open the obstruction under full visual control (Fig. 1D). In a case with concomitant frontal sinus mucocele, the frontal sinus was widely opened. A bicanalicular lacrimal tube was inserted (Fig. 1E) and finally, the nasal wound was packed with an alignment wound dressing.

Postoperatively, intravenous antibiotics were administered for 3 days and antibiotic and steroid eyedrops were administered. The lacrimal tube was removed 3 months after surgery.

## 3. Results

**Figure 2.**
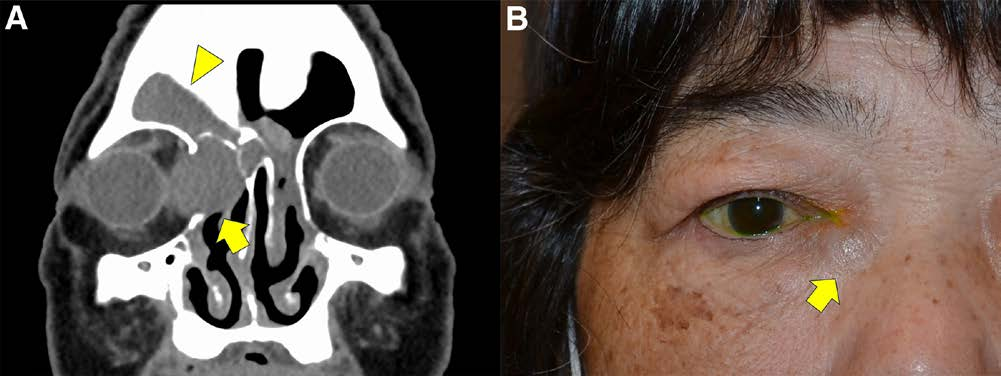
Clinical images. (A) A coronal computed tomographic image in Case #1 showing both lacrimal sac enlargement (arrow) and frontal sinus mucocele (arrow head). (B) A face photo in Case #2 showing a bluish lesion in the medial canthus (arrow).

Table 1 summarizes the demographic and clinical data of the patients. The study included 3 sides with nasolacrimal obstruction and acquired dacryocystocele from 3 individual patients. (age range, 68–88 years; 1 male and 2 females; 2 right-sided and 1 left sided; Table 1). Duration of symptoms including swelling in the medial canthal region, epiphora and discharge ranged from 2 weeks to 2 years. Case #1 had a history of nasal surgery on the same side of dacryocystocele and showed concomitant frontal sinus mucocele (Fig. 2A). Case #2 presented with bluish swelling in the medial canthal region (Fig. 2B).

**Table 1:** Patient characteristics.

Case #	1	2	3
Age	74	68	88
Sex	M	F	F
Side	R	R	L
Past medical history	Right nasal surgery 60 yr ago, DM	DM, heart failure	Right DCR 10 yr ago
Symptom duration	2 wk	6 mo	2 yr
Bluish swelling in the medial canthal region	Absence	Presence	Absence
Concomitant sinusitis	Frontal sinus mucocele	Nil	Nil
Common canalicular obstruction	Complete	Complete	Complete
Pathologic result	Fibroconnective tissue with columnar epithelium	Sac wall with squamous epithelium and goblet cells	–
Culture test result	MRCNS	S. aureus	MSSA

M = male, F = female, R = right, L = left, DM = diabetes mellitus, DCR = dacryocystorhinostomy, MRCNS = methicillin-resistant coagulase negative *Staphylococci*, MSSA = Methicillin-Susceptible *Staphylococcus aureus*, S. aureus = *Staphylococcus aureus*.

Dacryoendoscopic examination revealed complete obstruction at the level of the common canaliculus in all patients, just before the entry to the lacrimal sac. This was confirmed by the presence of structural occlusion, such as a whitish, fibrotic membrane, which created mechanical resistance to saline flow. Pathological analysis in Case #1 revealed fibroconnective tissue with columnar epithelium, while Case #2 showed a lacrimal sac wall lined with squamous epithelium and goblet cells. Pathological examination was not performed in Case #3. Culture test of lacrimal sac content showed methicillin-resistant coagulase negative *Staphylococci* in Case #1, *Staphylococcus aureus* in Case #2, and methicillin-susceptible *Staphylococcus aureus* in Case #3.

Surgery was successful in all cases with no intraoperative or postoperative complications. In all patients, nasal endoscopy confirmed that the rhinostomy site was widely open and fully epithelialized. All subjects reported complete resolution of symptoms with no recurrences observed at the 6-month follow-up based on clinical evaluation and endoscopic examination.

## 4. Discussion

In this study, we present for the first time the use of direct dacryorndoscopic probing in combination with endonasal DCR in the treatment of acquired dacryocystocele.

All dacryocystomucoceles require a blockage of the distal canaliculi.^[1]^ However, the role of proximal common canalicular obstruction remains controversial and is not yet fully understood, with most proposed mechanisms focusing on the congenital form.^[7]^ Some reported cases have demonstrated functional obstruction,^[1,6,8–10]^ while others showed no obstruction at all.^[6,8,11,12]^ Only few cases have been reported with mechanical obstruction identified by a soft stop during probing^[4,5,13]^ or under digital subtraction dacryocystograph.^[14]^ Previous studies suggested a functional obstruction due to a ball-valve effect at the common canalicular lacrimal sac junction, which inhibits reflux.^[9,11,14]^ Additionally, sac enlargement may result in canalicular displacement and, in some cases, compression or kinking.^[5]^ Chronic inflammation can also lead to swelling of Rosenmüller’s valve, impairing its function.^[2,5]^ In our series, all 3 cases exhibited complete common canalicular obstruction, as confirmed by direct observation using dacryoendoscopy. This finding suggests that proximal mechanical obstruction may be more prevalent than previously thought. Furthermore, it may also provide an explanation for cases of DCR failure in dacryocystocele patients with concurrent common canalicular obstruction when a canalicular tube is not used.

Although canalicular obstruction can be identified through regular probing, indicated by a soft stop, the dacryoendoscope offers additional value beyond diagnosis, playing a valuable role in treatment as well. The dacryoendoscope enables precise, visualized probing, offering a more reliable alternative to blind probing, which carries the risk of false passage formation.^[15]^ Treating common canalicular obstruction is crucial for resolving epiphora symptoms. In many cases, precise and direct visualization during probing can successfully open the obstruction, potentially reducing the need for conjunctivodacryocystorhinostomy.^[4]^ We believe that adequately addressing both proximal and distal obstructions can lower recurrence rates and improve the success of endonasal DCR surgery. In patients presenting with dacryocystocele, it is essential to assess common canalicular patency at the end of the endonasal DCR procedure. If an obstruction is present, we recommend performing probing in a controlled setting under direct dacryoendoscopic visualization.

This study has several limitations, including its retrospective design, small sample size, and short follow-up period. We recommend the use of direct dacryoendoscopy in future research to further evaluate the clinical value of this approach in a larger patient population. Prospective studies with a larger sample size are needed to confirm these findings.

In conclusion, acquired dacryocystocele is a rare condition that requires careful evaluation and appropriate management. Our findings suggest that proximal common canalicular mechanical obstruction may be more prevalent than previously recognized. Direct dacryoendoscopic probing provides both diagnostic and therapeutic advantages, allowing for precise visualization and effective treatment of canalicular obstruction. By addressing both proximal and distal obstructions, this approach may improve the success rates of endonasal DCR and reduce recurrence.

## Author contributions

**Conceptualization:** Yasuhiro Takahashi.

**Data curation:** Yasuhiro Takahashi.

**Formal analysis:** Yasuhiro Takahashi.

**Investigation:** Kinga Yo, Miho Enoki, Yuki Wasai, Yasuhiro Takahashi.

**Methodology:** Yasuhiro Takahashi.

**Project administration:** Yasuhiro Takahashi.

**Supervision:** Yasuhiro Takahashi.

**Validation:** Yasuhiro Takahashi.

**Writing – original draft:** Muhammad Abumanhal.

**Writing – review & editing:** Kinga Yo, Miho Enoki, Yuki Wasai, Yasuhiro Takahashi.
